# Amniotic membrane scaffold in conjunction with Biodentine for apexification: a clinical study on immature permanent teeth

**DOI:** 10.1186/s13104-025-07625-1

**Published:** 2026-01-19

**Authors:** M. Roma, M. Kundabala, Shreya Hegde

**Affiliations:** Department of Conservative Dentistry and Endodontics, Manipal College of Dental Sciences Mangalore, Manipal Academy of Higher Education, Karnataka, Manipal 576104 India

**Keywords:** Apexification, Immature tooth, Apical barrier techniques, Regenerative endodontic procedure, Amniotic membrane, Health, Well being

## Abstract

**Objective:**

In Endodontics, the primary objective of regenerative treatment is to revitalize the pulpo-dentinal complex of immature permanent teeth, promote continued root development, and achieve complete closure of the open apical terminus. Traditionally, apexification has been performed using conventional pulp space therapy for all non-vital immature permanent teeth. In this study, after rubber dam isolation and local anesthesia, routine endodontic procedures were initiated. Access cavity preparation was followed by biomechanical preparation using ProTaper hand files (size F3). Irrigation was carried out with 3% sodium hypochlorite, and two rounds of intracanal calcium hydroxide dressing were placed. At subsequent visits, the dressing was removed, the canals were dried with absorbent points, and an amniotic membrane scaffold (Tata Memorial Hospital, Mumbai, India) was positioned at the apex for two weeks. The entire pulp space was then obturated with Biodentin, and composite resin was used for post-endodontic restoration.

**Results:**

Apexification protocols were followed in ten adult non-vital permanent teeth with open apices. Clinical and radiographic evaluations demonstrated satisfactory healing. The amniotic membrane appeared to contribute to apical closure within two weeks and showed no adverse effects. It may represent a promising scaffold material for apexification in developing non-vital permanent teeth.

*Trial registration number:* Current Controlled Trials CTRI/2025/11/097852 [Registered on 21/11/2025].

## Introduction

The dental pulp sustains permanent impairment because of caries spread and trauma throughout odontogenesis. If they take place during the developing phases, the course of treatment can be modified to promote successful root end closure by considering the alterations in the tooth’s internal structure and having a solid grasp of the open apex. Approximately 30% of cases of necrotic pulp in the young permanent dentition are caused by trauma, which is commonly linked to open apices in immature permanent teeth [1].

Dental cavities and damage to immature adult teeth with an open apex pose a substantial threat to their functionality. Root formation stops when the pulp of a newly formed permanent tooth necrotizes. In certain situations, apical closure is not possible. These teeth typically have broad, open apexes and fragile root walls, which present several difficulties for the dentist doing root canal therapy, particularly on an adult permanent tooth. To prevent filling material from extruding when it is compacted in these gaping apices, it is also crucial to generate an apical closure. Calcium hydroxide is said to be probably the most archetypal substance for apexification. However, biomimetic materials such as MTA and Biodentine can be utilized as replacement components for apical closure.

Apexification can be defined as the continual development of the apical end of a young adult tooth with necrosed pulps or the initiation of a calcification superstructure in a permanent tooth with a gaping apex [2]. To prevent microorganisms and their toxic substances from moving from the pulp chamber to the periapical area, this approach aims to create a root apical barrier [3]. In contrast to MTA or Biodentine, which inclines to transmit more quickly, apexification with Ca(OH)2 takes a lengthy time. Hence, these bioactive materials have been used to the apical barrier in recent years. It likewise becomes difficult to insert these bioactive materials at the open apices, avoiding extrusion. Therefore, it would prove quite beneficial to have a proactive apex sealing barrier that encourages the apexification process.

Furthermore, regenerative branches of dentistry have stimulated the development of conductive and inductive biomaterials, among them bovine pericardium membranes, bioresorbable membranes, made of collagen, most frequently used in clinical routine. In the light of this evidence, amniotic membrane, biodentine, and bovine pericardiummembrane are complementary, due to the fact they can be used in different phases of regenerative endodontic therapy [2].

At the same time, regenerative endodontics and tissue engineering also includesother bioactive scaffolds, like amniotic membranes and dentin derivatives, consideredas natural, biologically rich matrices, while biodentine is a synthetic but bioactiverestorative material. These biomaterials, together, create a synergistic triad [3].

In the current era of regenerative healthcare, the apical sealing wall can be formed using amniotic membrane. The amniotic membrane, a connective tissue rich in stem cells, has been utilized as a restorative material in experiments. The innermost and thickest component of the placenta is the amniotic membrane, often known as the amnion. It is made up of a mesenchymal matrix with no circulation and a thick basement layer [4]. Reparative dentinogenesis may benefit from the amnion-derived units of the maternal membrane’s capacity for multifaceted diversification [5]. This membrane has received great popularity and is being applied to surgical operations and burn injuries [6]. It has been seen that the amniotic membrane promotes additional epithelialization, prevents the spread of infection, and reduces discomfort and inflammation [7]. Research and case investigations have illustrated that safety concerns arise with the use of amniotic membrane in the medical field [8]. Research has demonstrated that amniotic membrane treatment remains secure, efficient, and simple to use [9]. This membrane is readily available and ethically unproblematic [10]. Because it is capable of differentiation and has little immunologic activity and low potential for cancer, the amniotic membrane is an intriguing alternative to stem cells [11].

The development of mineralized tissue for root end closure is aided by the amniotic membrane’s implantation into the open apex. Only few clinical studies have evaluated the use of amniotic membrane for apexification, and the available evidence is limited to isolated case reports and small case series. This highlights the novelty and originality of the present study [12, 13]. Growth factors and cytokines found in abundance in the amniotic membrane can regulate inflammation and stimulate cementogenesis and odontogenesis. Its biological activity may help apical closure by maintaining Hertwig’s epithelial root sheath (HERS) and promoting the development of stem cells from the apical papilla (SCAP) into odontoblasts [14, 15].

Biodentine is a calcium silicate-based cement with optimal properties such as favorable biocompatibility, bioactive behavior, and excellent mechanical properties. It also possesses excellent sealing ability, high compressive strength, short setting time, and a pH above 12, which would confer favorable antimicrobial activity, making it an effective material for vital pulp therapy [11].

During the examination reviewing sessions, the radiologic diagnostics validates this. This cross-sectional longitudinal study aimed to document the clinical effectiveness and radiographic outcome of favorable therapeutic response in young permanent teeth employing amniotic membrane for the creation of the calcific bridge, followed by biodentin obturation, over a 12-month interval.

## Materials and methods

### Ethical approval

This study was authorized by the Institutional Ethics Committee of MCODS Mangalore under the IEC number 17,143. The CTRI registration of this prospective study was done under the number: CTRI/2025/11/097852.

### Study design and settings

This research was carried out in the Department of Conservative Dentistry and Endodontics, MCODS Mangalore. This cross-sectional study involved a novel single-step apexification technique using amniotic membrane as the scaffold and followed by complete obturation of the pulp space with Biodentine.

### Recruitment and eligibility criteria

The recruited subjects were selected based on the criteria for immature permanent anterior teeth with open apices. This exploratory cross-sectional study included 10 patients between the ages of 15 to 30 years with young permanent anterior teeth with necrotic pulps or apical periodontitis, who consulted the Department of Conservative Dentistry and Endodontics during the study period. Hence, the sample size could not be calculated. The primary data of the cases are included in Table 1. Individuals with immature anterior permanent teeth with AAE consensus of Pulp necrosis with/without symptomatic chronic apical periodontitis were recruited [16]. Radiographic signs of open apex root and periapical radiolucency were deferred to the principal investigator. Subjects who were deemed recalcitrant, had grossly decayed teeth, or with systemic disorders that affected their overall immunological condition were not allowed to participate in the study. The parents of the children who accepted to participate in the trial have been provided with all available details concerning the research project and provided consent in writing prior to treatment. The subjects who agreed to participate in the study have been provided with all available details concerning the research project and provided consent in writing prior to treatment.

### Blinding

The treating practitioner was unable to be blinded to the kind of material used to make the apical scaffold, as it was an interventional study. Yet, following completion of therapy as well as during inspections, the same practitioner did not participate in clinical and radiographic assessments. Two experienced investigators, who were blind to the type of material employed as an apical matrix and assessed the treatment outcomes.

### Clinical procedures

**Fig. 1:**
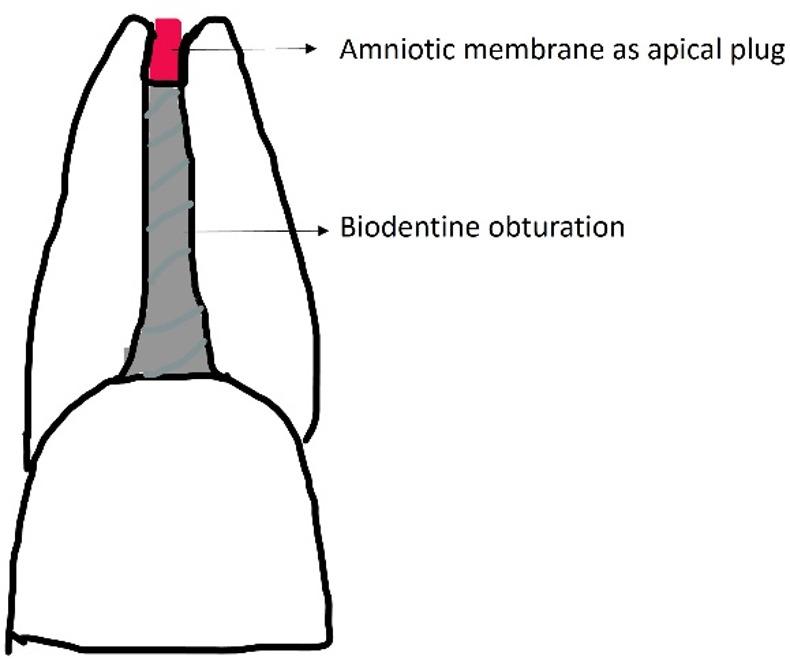
Diagrammatic representation of the amniotic apical plug followed by biodentine obturation

Pre-operative Intraoral periapical radiographs of the involved teeth and CBCT (Planmeca Promax 3D Mid, USA Inc) were taken to confirm the confirmation of open apex. Patients were informed about the treatment. Diagrammatic representation of the procedure is shown in Fig. 1.

A bur (carbide fissure) fitted on a handpiece with low speed was used to eliminate all cavities while rubber dam isolation and local anaesthesia were in place. Access cavities were prepared and refined with Endo-Z bur (Dentsply Maillefer, Tusla, OK, USA). 3% sodium hypochlorite (Vishal Dentocare Pvt Ltd, India) was used to copiously irrigate the pulp space. With endodontic K files (Dentsply Inc, Maillefer, Dentsply India) the working length was measured radiographically and positioned at 1 mm short of matching root apex. Cleaning, shaping and biomechanical debridement was achieved with hand K files and Protaper hand files up to size F3 (Dentsply Inc, Maillefer, Dentsply India). The treated pulp spaces were copiously irrigated with 3% sodium hypochlorite (Vishal Dentocare Pvt Ltd, India). The completed pulp spaces with sterile absorbent points were dried and subjected to intracanal medication using non-setting calcium hydroxide paste (CALCICURE, Voco, Germany) over a course of 2 weeks and provisionally sealed with intermediate restorative material (Cavit G, 3 M ESPE GmbH, Neuss, Germany).

**Fig. 2:**
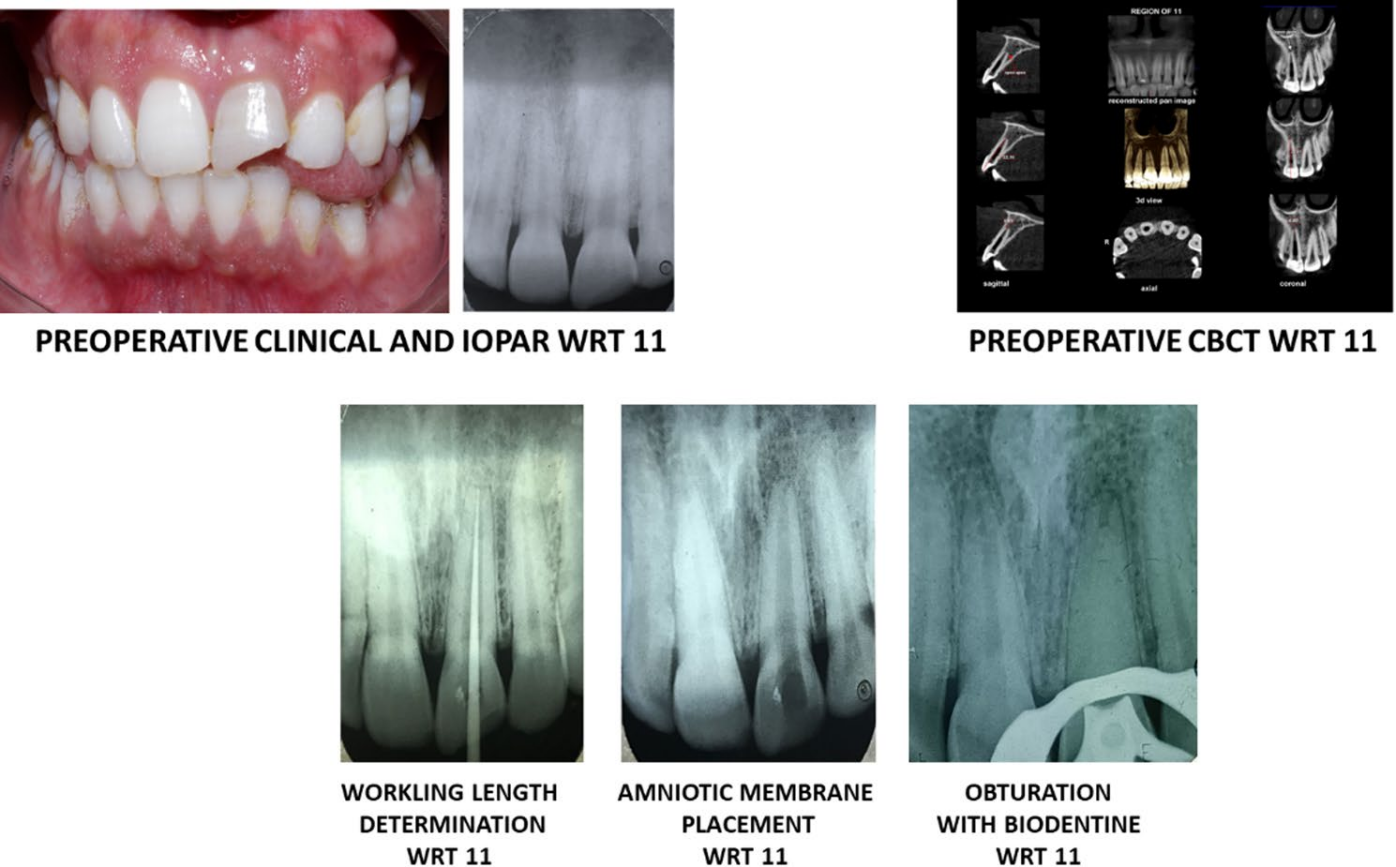
Clinical approach demonstrating the insertion of amniotic membrane as an apical plug, succeeded by obturation with Biodentine

In the subsequent visit, the treated tooth was assessed for any clinical signs and symptoms. The tooth was reentered, and the intracranial dressing was flushed out. Absorbent paper points blotted out excess saline. Using sterilized scissors, Amniotic membrane derivative (AMD) (Tata Memorial Hospital, Mumbai, India) was sliced as required, and was plugged at the apical terminus with the help of hand plugger (Dentsply Maillefer, Switzerland). The AMD was left in place for 2 weeks for the genesis of the apical barrier. Placement of the apical barrier membrane was confirmed with the help of IOPAR (Fig. 2). Salted saline was used for moisturizing the readily accessible processed freeze-dried irradiated human amniotic membrane (ACTREC, Tatanagar Memorial Hospital tissue bank, Mumbai, India). Using a finger plugger in the apical part of the canal, the barrier was gradually packed inside. With an ISO 9001:2000 Certified Quality Management System, ACTREC is the initial tissue bank in India to sterilise biological tissues using radiation. The American Association of Tissue Banks’ criteria are followed for processing amnion tissue at Tata Memorial Hospital [17].

**Fig. 3:**
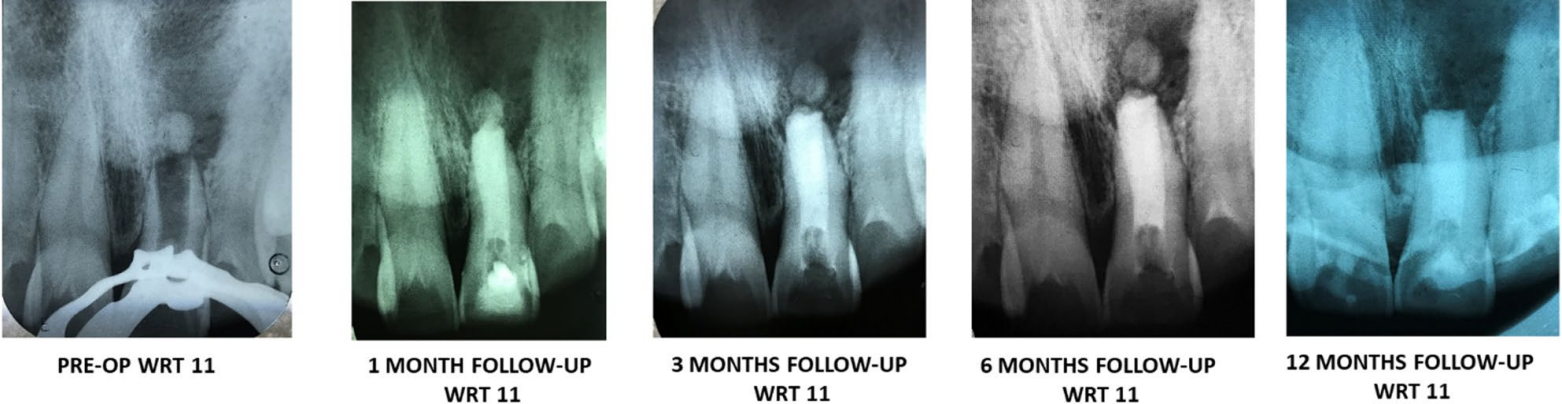
Follow-up with respect to 11

**Fig. 4:**
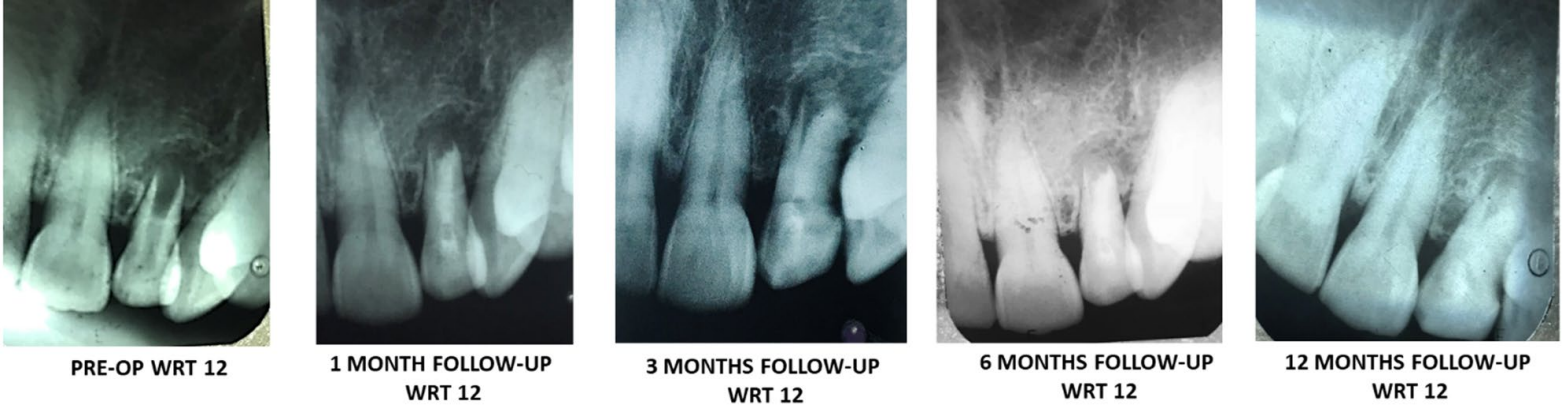
Follow-up with respect to 12

A periapical radiograph was taken to confirm the closure of the open apex prior to the start of the treatment. The canal was irrigated and dried followed by complete obturation with Septodont Biodentine^®^ (Lancaster PA, France) using MTA Endodontic Carrier (Dentsply Sirona Australia). Biodentine^®^ (Lancaster PA, France) is a di- & tr-calicum silicate material which acts a permanent dentinal substitute whenever the dentine is excessively damaged. Due to its hydroxyapatite deposition on the surface after its contact with the tissue fluids, the physical, mechanical and biocompatibility of Biodentine is considered as excellent material for apexification [14]. Figure 2 shows the schematic representation apexification of open apex using amniotic membrane derivative and Septodont Biodentine^®^ (Lancaster PA, France). To confirm the completion of the obturation, radiographs were collected at various intervals. Post endodontic sealing was achieved with composite resin (Filtek Z 350 XT, 3 M ESPE, St, Paul, MN, USA). The treated teeth were monitored at one-month, six-month, and one-year intervals (Figs. 3 and 4).

Follow-up clinical and radiographic assessment was carried out by two experienced practitioners, who were blinded to the study with the help of VAS score and PAI index criteria. Pain scores were assessed with the VAS score [18] and the PAI index score [19] were considered within normal range. Clinically, it was checked for tenderness on percussion, palpation, presence of mobility, and examined for any obscure swelling, sinus tracts, etc. Radiographic assessment involved apical root end closure, root genesis and mineralization, and recuperation of the periapical lesion. Management was deemed effective when the treated tooth showed no symptoms, had satisfactory periodontal health, and absence of periapical lesions in radiographs, with lamina dura continuity.

## Results

All ten cases treated with apexification procedure with amniotic membrane and Biodentin were prospectively followed over a period of 1 years (1 month, 3 months, 6 months, and 12 months) revealed signs of recovery with development of apical calcific bridge formation, healing of periapical lesions and absence of clinical symptoms. There was an absence of undue mobility, lack of tenderness to palpation, and percussion. The probing depths of the periodontist were normal. Normal bony architecture, preservation of apical seal, and continuous lamina dura were observed on radiograph. Visual Analogue scale and Periapical Index scores were deemed under normal. Table 1 enlists the treated and follow-up of clinical cases (anterior teeth) over a period of 12 months.

**Table 1:**
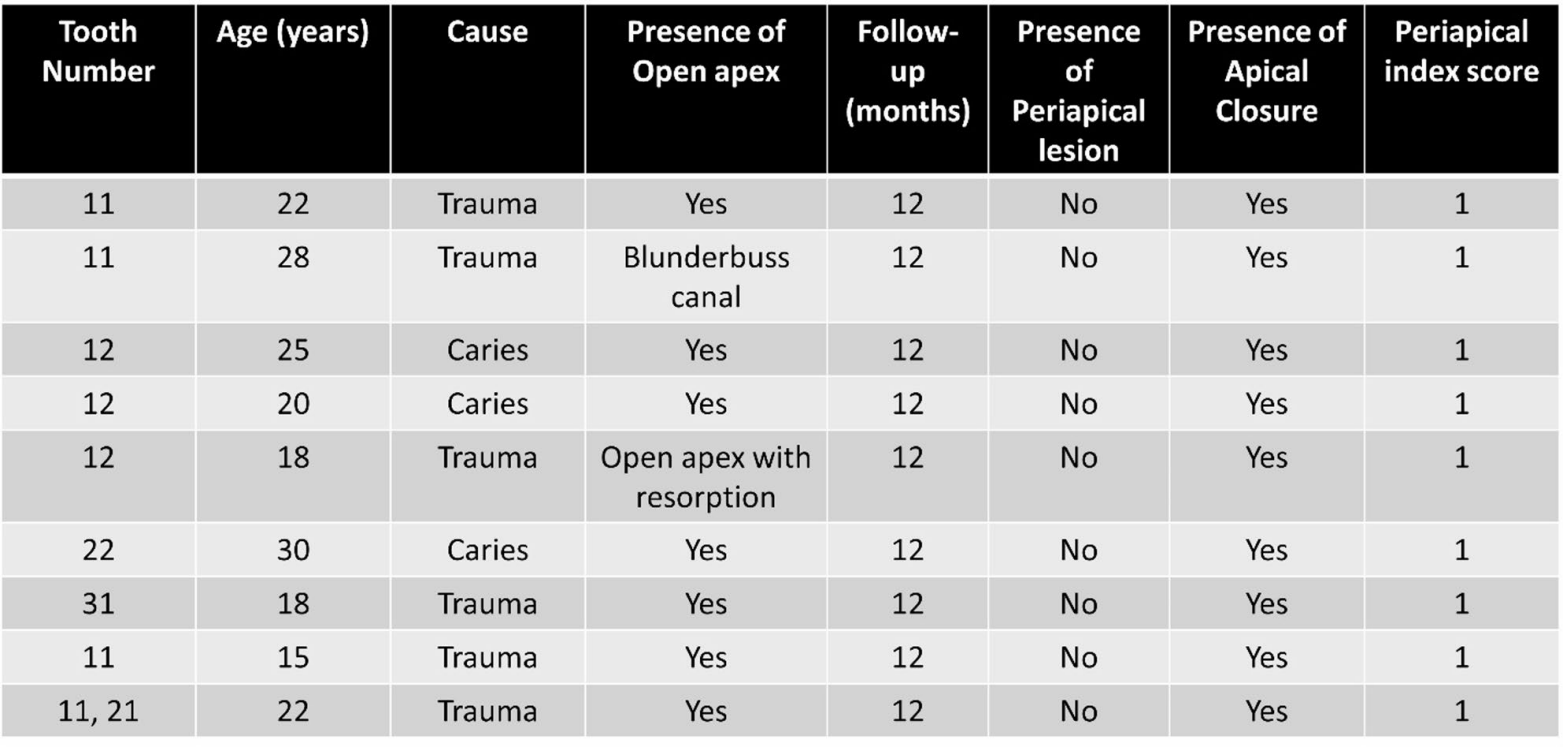
Primary data of various clinical cases

## Discussion

Hertwig’s Epithelial Root Sheath (HERS) is a pivotal element in root growth [20–23]. The cells of HERS emit preliminary impulses for the differentiation of SCAP (Stem cells in apical papilla) into odontoblasts, facilitating dentinogenesis on the root surface [24]. The HERS contributes to cementogenesis, hence facilitating the improvement in root morphology, and thereby creating apical closure [11, 12, 25]. Various clinical trials have been tried and tested to provide the basis forbiological rationale and links to the clinical outcomes of various materials in dental field (MTA, Ca(OH)₂, PRF, collagen scaffolds) [25–27].

Traumatic exposure at the earliest stages inhibits root development in young permanent teeth, leading to necrotic pulp and ensuing apical periodontitis. Periapical inflammation causes the release of markers linked to inflammation, including TNF alpha and IL-1, which disrupt the cellular functions of HERS and SCAP and impede the root’s ability to grow naturally [26, 27]. The functional growth of the root can be preserved by managing the periapical spread of infection, allowing the cells of the apical papilla and HERS to regain biological function and subsequently differentiate into odontoblasts [11, 25, 28]. Recent endodontic therapies that use synOss putty, which contains a collagen framework, have demonstrated positive outcomes in alleviating inflammation in the periapical region and facilitating apical barrier building, thus restoring the tooth’s functionality [29, 30]. In the current example, the foetal barrier, which is pluripotent and biologically active, facilitated root apex sealing and restored the expanded periapical periodontal ligament space through preserving Hertwig’s epithelial root sheath cells, so aiding in the reduction of periapical inflammation [31, 32].

The human fetal barrier is an excellent reservoir of stem cells with multiple potentials that aid in the formation of the root dentin and apical calcific bridge, as well as their transformation into odontoblasts [33]. The amniotic fluid contains cytokines that promote regenerative ability. This regenerating potential has been extensively studied in research studies, concluding that it aids in the healing procedure associated with persistent lesions [34–38]. The foetal membrane is extensively utilised in medical engineering as a framework material because to its minimal immunology and advantageous biological characteristics [39–42]. The characteristics of the amnion have surely contributed to the favorable outcome of our treatment plan.

The onset of haemorrhage into the pulp chamber space from the periapical region as a framework may interfere with HERS and hinder physiological root formation [43, 44]. Consequently, the advent of regeneration materials such as amnion eliminates the necessity for causing hemorrhage. In current instances, the pulp space disinfection with 3% NaOCl and Ca(OH)_2_ dressing should have effectively sterilised the canal and mitigated unsuccessful attempts attributable to getting infected.

In many apexification techniques, root completeness is achieved; nevertheless, maturing may remain incomplete for situations with an open apex. These outcomes have recently been examined in particular research studies and clinical investigations [17, 45]. Additional research is required to assess root completeness, and histological analyses must be conducted to investigate the nature of the tissue constituting the calcific bridge. Benatti et al. stated in their investigations that cemental tissue was collected on the canal peripheries and bony tissue was developed at the apical terminus [46]. Comparable structures have been identified in additional instances of open apex with apical periodontitis [47, 48]. The apical seal in this situation is ascribed to the function of the amnion in conjunction with HERS cells.

The application of bioceramic materials, such as Biodentine, as an obturating substance enhances root fortification and restores tooth functionality. This aligns with an investigation conducted by Girish et al., which indicated that complete obturation of the pulp area with Biodentine enhances the resilience to fracture of juvenile teeth in comparison to apexification groups [49]. The attributes of Biodentine, such as poor solubility, strong compressive ability, and decreased duration for setting (9–12 min), render it optimal for obturation. Another characteristic of Biodentine that renders it suitable for the obturation of juvenile teeth is its increasing compressive strength over time [50]. The compressive force of Biodentine reaches 300 MPa shortly after a single month, which is comparable to the compressive strength of normal dentine [51–53]. This concept renders Biodentine an optimal substance for the obturation of juvenile teeth; hence, we have integrated it accordingly. However, future perspectives should emphasize the need for randomized controlled trials, longer follow-up, and histological validation. Table 2 enlists various studies across the globe utilizing various biomaterials for endodontic regenerative procedures.

**Table 2:**
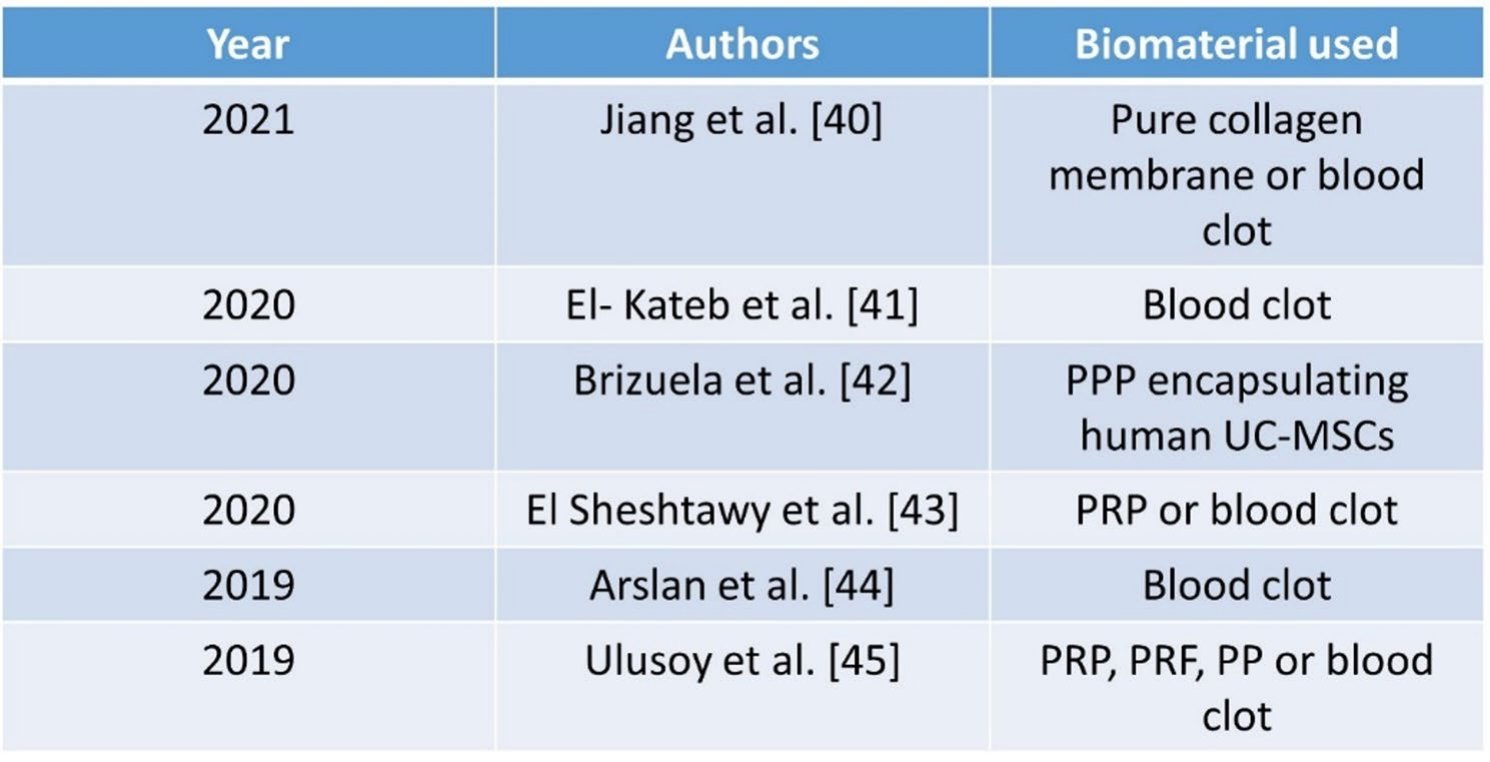
Various studies enlisting the utilization of various biomaterials for endodontic regenerative procedures

## Conclusion and limitations

The use of amnion as an apical plug, along with Biodentin as the obturating material, constitutes an effective apexification strategy for achieving apical seal of open apices. This technique not only offers superior sealing but also enhances the strength of nonvital teeth compared to conventional obturation methods. However, small sample size, absence of control group, no power analysis was performed, possible selection bias, limited reproducibility of membrane preparation and absence of histological validation are some of the limitations of the present study. But this technique can be considered as an alternative to the conventional therapy modalities as it helps in achieving apical seal with the use of regenerative scaffolds.

## Data Availability

Yes (The data that support the findings of this study are available from the corresponding author upon reasonable request.)

## Abbreviations

HERS: Hertwig’s Epithelial Root Sheath
SCAP: Stem Cells in Apical Papilla
AAE: American Association of Endodontists
CBCT: Cone Beam Computed Tomography
AMD: Amniotic Membrane Derivative
IOPAR: Intraoral Periapical Radiograph
